# Implementation of systems saving lives in Greece

**DOI:** 10.1016/j.resplu.2023.100358

**Published:** 2023-01-18

**Authors:** Anastasios Stefanakis, Theodoros Kalyvas, Nadine Rott, Bernd W. Böttiger, Evangelia Sigala

**Affiliations:** KIDS SAVE LIVES - Ta Paidia Sozoun Zoes, ERC CO, EMT Chalkidiki, Greece; ILS Provider Athens, Greece; Department of Anaesthesiology and Intensive Care Medicine, University Hospital of Cologne, Germany; Department of Anaesthesiology and Intensive Care Medicine, University Hospital of Cologne, Germany; Cardiac-Surgery Department, Hippokration Hospital of Athens, Greece

*To the Editor,*

Greece is among the countries with the lowest survival rate for out-of-hospital cardiac arrest (OHCA) victims in Europe, largely due to a low rate of lay resuscitation and a significant deficit in Cardiopulmonary Resuscitation (CPR) - First Responders (FRs).^1^

This fact prompted the founding of the Humanitarian Organization “KIDS SAVE LIVES - Ta Paidia Sozoun Zoes” (https://kidssavelives.gr/en/home/) in Greece (2016, Scientific Directors A. Stefanakis and A. Samaras). The organization (with pilot action since 2010) has created community-level programs aimed at providing continuous education in CPR and a national lifesaving network aligned with the System Saving Lives guidelines.^2^ Greek schools have been a key area for this and after having received approval from the Ministry of Education (2017), we have started CPR training under the umbrella of the international movement KIDS SAVE LIVES (KSL).^3^ To date, 53.793 students and teachers have been trained. Also, 27.000 pupils received online training during the COVID-19 pandemic. These goals were achieved through the contribution of 826 volunteer European Resuscitation Council (ERC) instructors and the collaboration with the “Hellenic Society of Emergency Prehospital Care” (https://eeepf.gr/). Also, in 2021, the “KSL Lab Skills” were activated throughout the country. These concerned the training of students by their teachers, preparing the ground for the institutionalization of CPR in Greek schools in the official curriculum (2019, a proposal submitted to the Ministry of Education for approval). The next program soon to be implemented in Greek and European schools is LIFEFORCE (Learning Initiative For Elementary school Fun-Oriented Resuscitation Coaching Europewide), which enables pre-training of 6–10 year old school children in Basic Life Support and first aid).

Another area that received emphasis was the proliferation of Automated External Defibrillators (AEDs), with >300 AEDs placed in schools and communities by KSL-students. A National AED Map was created, which includes 1,233 verified operational AEDs (https://kidssavelives.gr/en/aed/). These are part of a community-implemented “FIRST RESPONDER SYSTEM” program that activates nearby certified volunteers in the event of a life-threatening situation. Also, the organization has been actively participating (2018) in the World Restart a Heart Day (WRAH) action, conducting several nationwide trainings simultaneously, an action that is also reflected in the System Saving Lives algorithm.^4^, ^5^ Within this framework, citizens and a large part of the student body are continuously trained in CPR at our official ERC training center. As a result, 97.000 children and adults have been trained, 5.650 and 456 of which have become Basic Life Support providers (BLS) and BLS Instructors (BIC), respectively. The latest innovation is the development and launch of the free iSAVElives application (https://kidssavelives.gr/en/isavelives/) which contains all the information needed to help in an emergency and to notify the Ambulance Service and FRs within a 10 km radius.

**Fig. 1:**
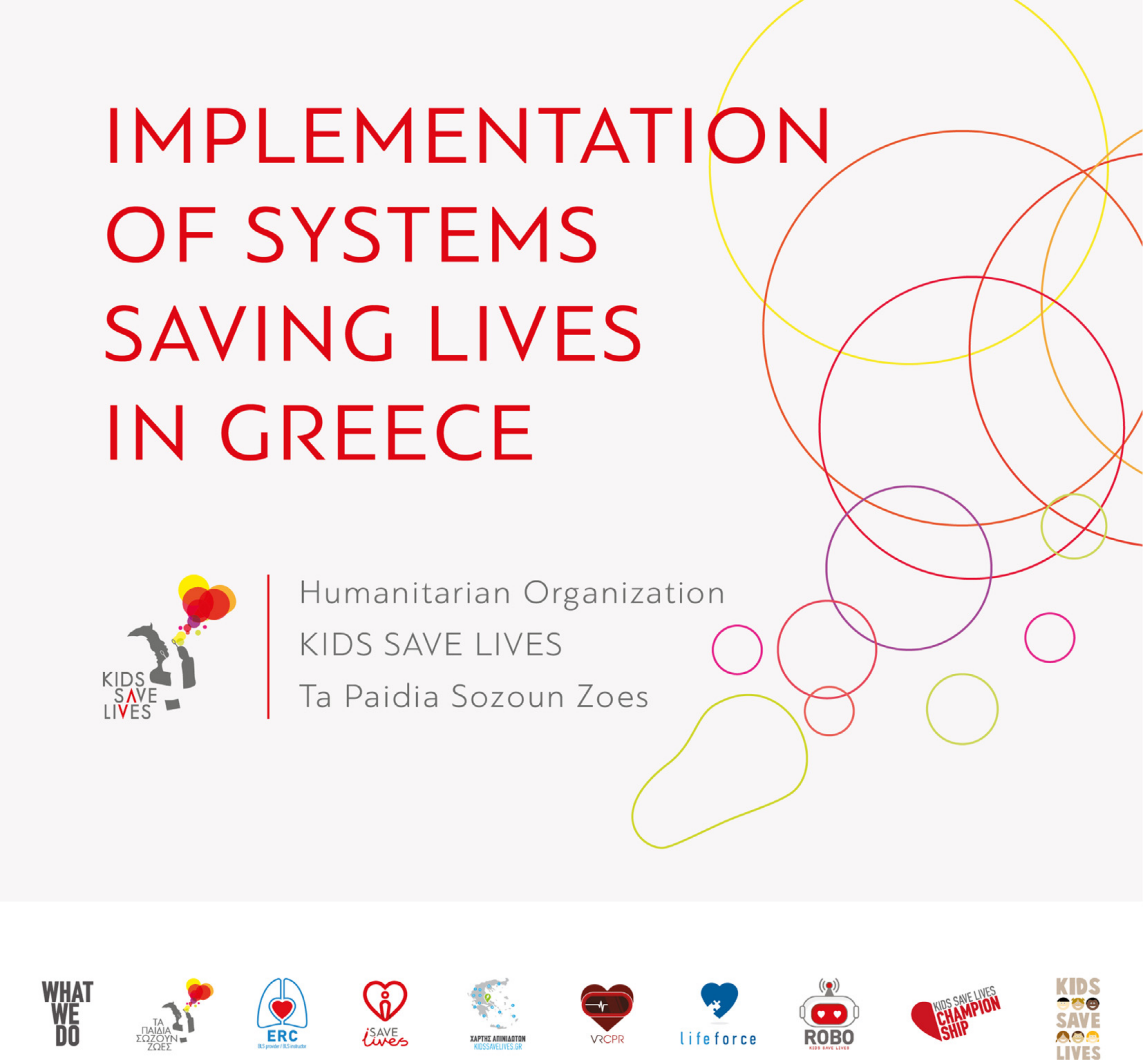
Logos of the Humanitarian Organization “KIDS SAVE LIVES - Ta Paidia Sozoun Zoes”.

Our actions (Fig. 1) had a positive impact on survival: 28 people who were in OHCA were successfully resuscitated and >300 life-saving situations were successfully treated. Efforts continue towards institutionalization of CPR as an official course in Greek schools.
